# A comparison of treatment outcome between fiducial-based and bone-based image guided radiotherapy in prostate cancer patients

**DOI:** 10.1186/s13014-018-1171-2

**Published:** 2018-11-27

**Authors:** Aleksandra Napieralska, Wojciech Majewski, Roland Kulik, Grzegorz Głowacki, Leszek Miszczyk

**Affiliations:** 1 Radiotherapy Department, Maria Sklodowska-Curie Memorial Cancer Centre and Institute of Oncology, Wybrzeze Armii Krajowej 15, 44-100 Gliwice, Poland; 2 Radiotherapy and Brachytherapy Planning Department, Maria Sklodowska-Curie Memorial Cancer Centre and Institute of Oncology, Gliwice, Poland

**Keywords:** Prostate, Radiotherapy, Image-guided, Fiducial

## Abstract

**Background and purpose:**

To compare the clinical outcome in prostate cancer patients treated with radiotherapy using two forms of image guidance: bone-based (BB) or fiducial-based (FB).

**Material and methods:**

This retrospective study consisted of 180 patients treated with kV-kV image-guided radiotherapy (IGRT) between the years 2008 and 2011. A total of 89 patients were aligned to pelvic bone (Group BB) and 91 patients to the fiducial implanted into prostate for image guidance (Group FB). Patients were treated to a total dose of 76 Gy in 38 fractions. The Cox Regression Model was used to evaluate the influence of clinical and treatment-related parameters on overall survival, biochemical progression and progression-free survival. Acute and late toxicity were evaluated based on the RTOG/EORTC criteria. Sexual function was assessed with QLQ PR-25 (EORTC QLQ forms). An assessment of the differences in patient daily set-up from the time of simulation was performed.

**Results:**

The incidence of acute G2/G3 genitourinary (GU) and gastrointestinal (GI) toxicity was similar between groups. In the BB group, 34 patients had G2 and 5 had G3 GU acute toxicity - compared to 40 patients with G2 and 2 with G3 in the FB group. G2 and G3 GI acute toxicity was observed respectively in 24 patients and in 1 patient in the BB group compared to 18 patients with G2 and 1 patient with G3 toxicity in the FB group. The five-year incidence of late ≥G2 GU toxicity was 12% in both groups (*p* = 0.98) and ≥ G2 GI toxicity 19% (BB) vs 15% (FB, *p* = 0.55), respectively. The five-year progression-free survival rate was 87% in BB and 81% in the FB Group (*p* = 0.15). The 5-year Overall Survival rate (OS) was 80% (BB) vs 91% % (FB, *p* = 0.20), but the difference was most pronounced in the intermediate-risk group: 5-year OS of 93% (FB) and 75% (BB), respectively (*p* = 0.06). No significant changes were observed in sexual or erectile functioning as compared to that specified at the beginning of radiotherapy and between the FB and BB Groups.

**Conclusion:**

When comparing bone-based to fiducial-based techniques, no differences in clinical outcomes or late toxicity were seen in this population. However, intermediate risk prostate cancer patients are those who might benefit most from implementation of fiducial-based IGRT.

## Background

In recent years, many different image-guidance systems have been developed in order to improve daily on-line corrections of interfraction translational prostate displacements [1–6]. These strategies have enabled the possibility of considerable improvement in radiation effectiveness in terms of a potential decrease in side effects whilst increasing the dose to the tumor. One of the image-guided radiotherapy (IGRT) techniques is based on the use of fiducial gold seeds implanted into the gland and a pair of kilovoltage (kV) images taken daily for the patients’ setup. The use of fiducials allows not only for visualization of the exact position of a prostate gland, but also for a significant reduction in radiotherapy planning margins [6–9]. Before the implementation of markers, bony matching techniques were used daily with two dimensional - planar orthogonal kV radiographs [3, 4, 6, 10–12]. Other methods of IGRT include the use of ultrasound, megavoltage X-ray imaging, electromagnetic tracking, MRI or cone beam computed tomography [13–15]. Despite the importance of IGRT, there are only a few studies which have compared different methods of IGRT techniques in relation to the patients’ treatment outcome [16–20]. In order to evaluate the potential influence of fiducial implantation on the treatment results, we compared two groups: irradiated with bone-based or fiducial based IGRT.

The aim of this study is to compare clinical outcomes - acute and late toxicity, overall and progression-free survival in patients with prostate cancer treated with radiotherapy (RT) using one of the two forms of image guidance: bone-based (BB) or fiducial-based (FB).

## Methods

### Patients characteristics

The retrospective study group consisted of 180 patients treated with curative-intent RT between the years 2008 and 2011 at the Radiotherapy Department of the Maria Skłodowska – Curie Memorial Cancer Centre and Institute of Oncology in Gliwice. The policy of IGRT changed during this period due to the availability of fiducials for image guidance from approximately 2009. The inclusion criteria are presented in Table 1.

**Table 1:** Study inclusion criteria

Inclusion criteria	
Histopathological confirmation of prostate cancer Indication of Gleason score and the highest value of prostate-specific antigen (PSA) concentration Clinical stage assessment according to the TNM classification No distant metastases (M0) confirmed based on abdominal ultrasound/CT, chest X-ray/CT and scintigraphy No other concurrent or previous cancer (excepting skin cancer, with at least a 5-year disease free follow-up) Radiotherapy as a definitive treatment method, using uniform contouring criteria of treatment volumes and critical structures; 3D or dynamic techniques; image guided radiotherapy (IGRT) Regular (weekly during treatment and on every visit during follow-up) evaluation of acute and late toxicity from the gastrointestinal and genitourinary tracts, based on the EORTC/RTOG criteria	

Information regarding patient and treatment characteristics was collected from the patients’ charts and from the treatment planning system files. Data were collected based on the following parameters: the date of prostate cancer diagnosis, age, performance status, Gleason score, tumour stage, PSA concentration (at the time of diagnosis and during follow-up), primary treatment (IGRT method, doses used), the date of biochemical or pathological failure after primary treatment, the number and type of metastases (bone/lymph node), data concerning hormonal therapy, the date of local control, progression outside the treated field, biochemical progression and death, acute and late genitourinary and gastrointestinal toxicity, sexual dysfunction and co-morbidities. In the univariate and multivariate analysis, all these factors were analyzed with respect to the study end-points. Treatment planning was performed using the Eclipse system and patients were treated with linear accelerators. All patients had online kV-kV image guidance every day. The differences in patient daily set-up from the time of simulation were collected in terms of vertical, longitude and lateral directions. The data was statistically evaluated as relative and absolute differences between the BB and FB Groups. All data were collected as relative (with respect to shift direction) and as absolute values (which represent a value of the shifts irrespective of direction). A 0 value represents the identical position of the patient as during treatment planning. The U Mann Whitney test was used to evaluate the significance of differences between groups.

A total of 180 patients with prostate cancer were treated with curative-intent RT during the study period. The age of patients at the time of the treatment ranged from 49 to 85 years (mean 69, median 70). All of them presented as in a good performance status. The patients’ detailed characteristics are presented in Table 2.

**Table 2:** Clinical features of irradiated patients

Bone – based image guided group (BB) *N* = 89		Fiducial – based image guided group (FB) *N* = 91		
Characteristics	Number (%)	Characteristics	Number (%)	*p*
T stage		T stage		0.93
1	36 (41%)	1	39 (43%)	
2	42 (47%)	2	38 (42%)	
3	8 (9%)	3	13 (14%)	
4	3 (3%)	4	1 (1%)	
Gleason score		Gleason score		
6	56 (63%)	6	43 (47%)	0.66
7	23 (26%)	7	32 (35%)	
8	7 (8%)	8	10 (11%)	
9	1 (1%)	9	6 (7%)	
10	2 (2%)	10	0 (0%)	
Highest PSA		Highest PSA		
Range (ng/ml)	3.41–150.00	Range (ng/ml)	1.97–143.00	0.36
Median	14.50	Median	14.12	
Mean	27.31	Mean	22.84	
Risk group		Risk group		
Low	19 (21%)	Low	13 (14%)	0.84
Intermediate	32 (36%)	Intermediate	42 (46%)	
High	38 (43%)	High	36 (40%)	
Hormonal therapy		Hormonal therapy		
Yes	77 (87%)	Yes	81 (89%)	0.61
No	12 (13%)	No	10 (11%)	
Symptoms		Symptoms		
*Urinary*		*Urinary*		0.01
Yes	42 (47%)	Yes	27 (30%)	
No	47 (53%)	No	64 (70%)	
*Intestinal*		*Intestinal*		0.45
Yes	5 (6%)	Yes	3 (3%)	
No	84 (94%)	No	88 (97%)	
Cardiovascular disease		Cardiovascular disease		0.01
Yes	46 (52%)	Yes	64 (70%)	
No	43 (48%)	No	27 (30%)	

As it was not a randomized study, some imbalance with respect to cardiovascular diseases and urinary symptoms before radiotherapy was observed, although this was not associated with any change in the method of assessment of such symptoms. There were no statistical differences between the groups regarding other clinical factors such as: T-stage, PSA concentration, Gleason score etc. The low (LR), intermediate (IR) and high-risk (HR) groups were observed in group BB and FB in 21 and 14%; 36 and 46%; 43 and 40%, respectively (*p* = ns).

The majority of patients started hormonal therapy (HT) before RT. The median PSA concentration at the start of RT was 0.683 ng/ml (range: 0.003–40.346).

### Treatment planning

An individual thermoplastic mask covering the pelvic region in the supine position was made for each patient. A CT of the pelvic and abdominal regions was conducted with 3–5 mm slices, 1.5–2 h after drinking 250–500 ml of water. The planning target volume (PTV), clinical target volume (CTV) and organs at risk (OARs) were contoured by one clinician. The rectum was contoured from the rectosigmoid junction to the anus. The intestines were contoured from the level of the L5 vertebra down to the rectosigmoid junction. The bladder was contoured along the external border of the organ.

A total of 89 patients were positioned to pelvic bones (Group I - BB) and 91 patients to one fiducial (Group II - FB) (GoldAnchor™) implanted inside the prostate. All FB patients were implanted with one fiducial 20 mm in length and 0.3 mm in diameter. Fiducials are incised at each 2 mms to ensure stability after implantation. The procedure was performed transrectally, using an ultrasound head with a specially designed guide and a 203 mm long (diameter 0.71 mm) needle. Implantation was performed 1 week before taking planning CT to stabilize the position of the marker.

### Radiotherapy

The patients were treated with 6–20 MV photons with a fraction dose of 2 Gy to the total dose (TD) of 76 Gy (38 fractions) to the prostate (apart from 4 LR patients from the initial stages of the study who received 74 Gy in 37 fractions of 2 Gy). The TD was delivered to the PTV which comprised of CTV (prostate ±1/2 of seminal vesicles) and an additional margin (7 mm in posterior and 9 mm in other directions). The patients in the HR group were treated with an elective pelvic lymph nodes irradiation to a TD of 44 Gy (fraction dose of 2 Gy). In those patients, a TD of 44 Gy was delivered to nodal PTV, which comprised of nodal CTV (pelvic lymph nodes: internal iliac, external iliac, presacral and obturator) and an additional margin (5 mm in all directions). The dose constraints for healthy tissues during the treatment planning are presented in Table 3.

**Table 3:** Dose-constraints for organs at risk

Organ	Dose [Gy]	Volume [%]
Rectum	70.0	20
	60.0	35
	50.0	50
Bladder	70.0	20
	60.0	35
	50.0	55
Bowel	40.0	30
Femoral heads	50.0	45

The majority of the patients (93%) were treated with dynamic techniques (IMRT, RapidArc) – 95% in the FB group and 91% in BB. The others were irradiated with 3D conformal techniques.

### Statistical analysis

Overall survival (OS) was evaluated using the Kaplan-Meier method. To verify the significance of variables influencing OS in the univariate analysis, the Cox proportional hazard model was employed. A *P*-value of less than 0.05 was considered indicative of statistical significance. Follow-up (FU) was defined as the period of time from the date of the beginning of RT to the date of the last visit, or death. Progression-free Survival (PFS) was measured from the date of the beginning of the treatment to the date of biochemical, local, distant progression, or death. Local control (LC) was defined as no progression within the prostate gland. The Phoenix criteria were used in defining biochemical progression – prostate specific antigen (PSA) nadir + 2 ng/ml. The differences in survival endpoints were calculated utilising the log-rank test. Calculation of the differences between the treated groups with respect to various factors was undertaken using the U Mann Whitney test or χ2 test. The study was performed according to the Helsinki Declaration and the Institutional Review Board Committee.

### Toxicity assessment

Acute and late toxicity was evaluated based on the RTOG/EORTC criteria [21]. All patients were examined before the start of RT and the intensity of their symptoms was noted as a reference point to grade the treatment toxicity. An evaluation was performed once a week during RT and on every visit during FU (every 3–4 months). Sexual function was assessed by means of QLQ PR-25 forms [22]. This was performed on all patients who had filled in the QLQ forms prior to RT and at 3–4 years follow-up and on those who had responded to questions regarding sexual activity and functioning. During this analysis we focused on the two most pertinent questions selected from the QLQ form: ‘To what extent were you sexually active?’ and ‘Did you have difficulty having or maintaining an erection?’ (with response options: ‘not at all’, ‘a little’, ‘quite a bit’, ‘very much’).

## Results

### Evaluation of positioning

An evaluation of daily differences in vertical, longitude and lateral directions in patient set-up from the time of simulation was performed. The median number of treatment days was 38 and the median number of days with IGRT was 36. In total 18,477 measurements (6159 days of treatment) were included in the statistical analysis. The value of the minimum, maximum, mean and median differences during treatment days, as well as standard deviation, were calculated by analyzing all shifts for each patient. The differences between the groups are presented in Table 4. The U-Mann Whitney test showed that the differences were statistically significant in terms of absolute mean (*p* = 0.00), absolute median (*p* = 0.00), absolute maximum (*p* = 0.00), absolute standard deviation (*p* = 0.002), relative minimum (*p* = 0.002) and relative standard deviation (*p* = 0.00) in favour of the FB group. The shifts were larger in the FB group, meaning that BB was less accurate. Hence, these results show that even the use of one fiducial better correlates with the position of the prostate during treatment than the positioning to the pelvic bone anatomy.

**Table 4:** Differences in daily patient set-up from the time of simulation in Fiducial-Based (FB) and Bone-Based (BB) Groups

Difference	FB group	BB group	FB group	BB group	FB group	BB group	FB group	BB group	FB group	BB group
	mean	mean	median	median	minimum	minimum	maximum	maximum	SD	SD
Absolute mean	0,39	0,27	0,38	0,25	0,16	0,00	0,83	0,65	0,15	0,13
Absolute median	0,28	0,16	0,30	0,10	0,10	0,00	0,70	0,50	0,13	0,12
Absolute maximum	2,09	1,67	2,00	1,70	0,80	0,00	5,80	3,50	0,74	0,59
Absolute SD	0,41	0,34	0,38	0,33	0,20	0,00	0,88	1,04	0,14	0,15
Relative mean	−0,01	0,02	0,00	0,02	-0,67	-0,38	0,54	0,45	0,20	0,15
Relative median	0,01	0,01	0,00	0,00	-0,60	-0,30	0,30	0,40	0,13	0,10
Relative minimum	−1,71	−1,32	−1,60	−1,30	−5,80	−3,50	−0,20	0,00	0,86	0,67
Relative maximum	1,57	1,38	1,40	1,40	0,20	0,00	4,00	3,20	0,68	0,61
Relative SD	0,54	0,42	0,51	0,41	0,25	0,00	1,11	0,91	0,18	0,16

^*^SD standard deviation

### Follow-up: Overall survival

**Fig. 1:**
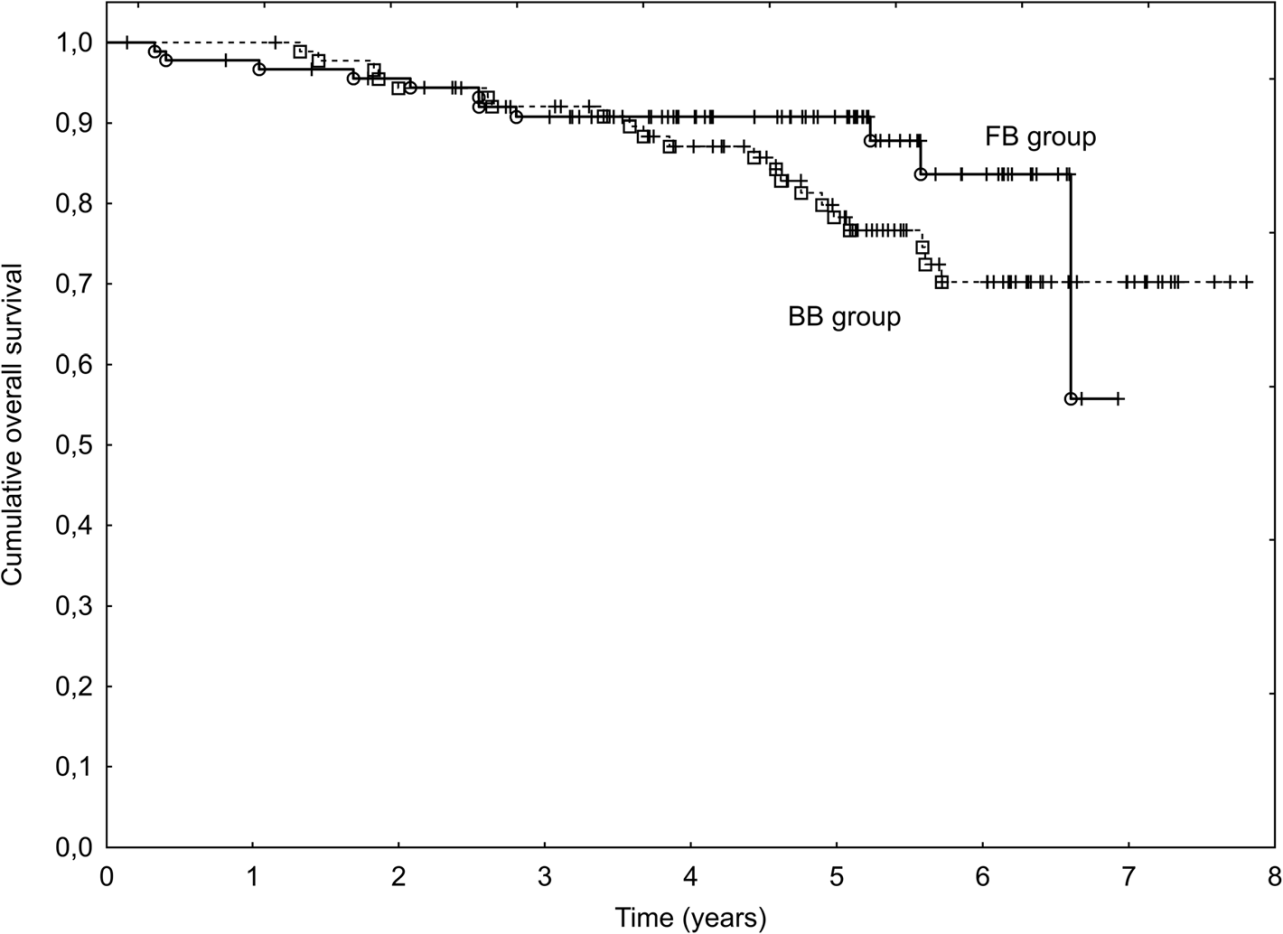
Overall survival in Fiducial-based (FB) and Bone-based (BB) IGRT groups

**Fig. 2:**
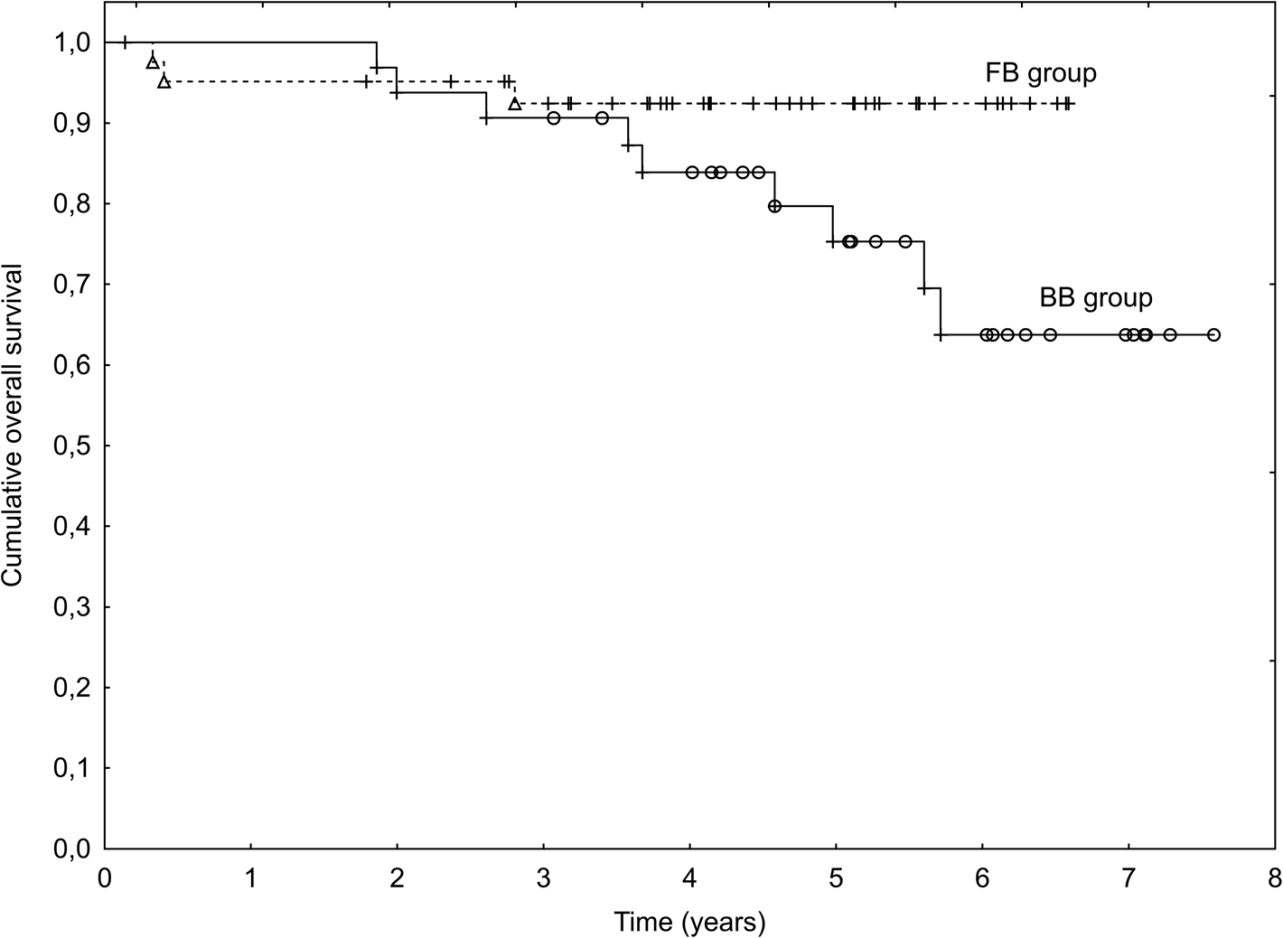
Overall survival in Fiducial-based (FB) and Bone-based (BB) IGRT groups – intermediate-risk (IR) patients (*p* = 0.062)

The median follow-up (FU) was 5 years during this time 32 patients died. The 5-year OS rate was 80% in the BB Group and 91% in the FB Group (*p* = 0.20), however the survival curves started to diverge after 4 years, favoring FB IGRT (Fig. 1). The difference was the most pronounced in the IR group – a 5-year OS of 93% (FB) and 75% (BB), respectively (*p* = 0.062). The results are presented in Fig. 2. According to the log rank test, patients without cardiovascular diseases (CVD) lived longer (a 5-year OS of 92 and 78%, respectively; *p* = 0.025). This observation particularly concerned patients in the FB group – a 5-year OS of 100% for those without CVD compared to 87% for those with CVD, *p* = 0.025. the difference in the BB group did not reach a level of statistical significance yet was also observed – 87% for patients without CVD compared to 70% with CVD, respectively (*p* = 0.103). In the multivariate Cox analysis: age (*p* = 0.001) and the presence of metastases (*p* = 0.030) were significant, independent factors influencing OS.

### Follow-up: Local control and progression free survival

**Fig. 3:**
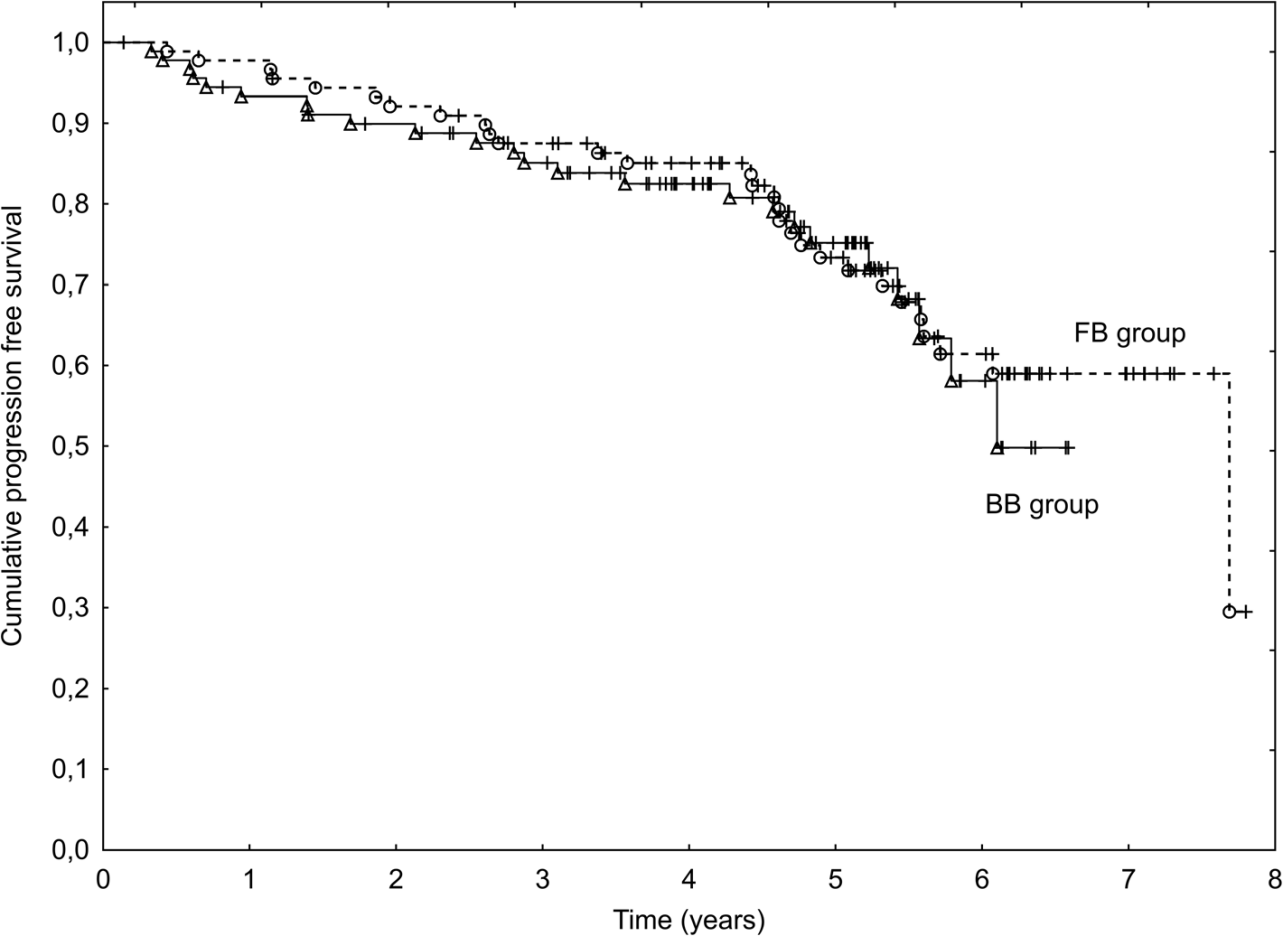
Progression free survival in Fiducial-based (FB) and Bone-based (BB) IGRT groups

Local failure was observed in 3 patients and the 5-year LC was 98% (2 recurrences in the BB Group and 1 in the FB Group). There was no statistical difference in PFS between the FB and BB Groups (*p* = 0.72) and the 5-year PFS was 75 and 73%, respectively (Fig. 3). Factors found to have a negative influence on PFS in the univariate analysis were: T-stage (*p* = 0.038), Gleason score (*p* = 0.005), age (0.039), the highest PSA concentration before RT (*p* = 0.032) and the risk group (*p* = 0.010). The multivariate analysis showed that only the Gleason score was an independent, significant factor influencing PFS (*p* = 0.050).

### Follow-up: Failure outside irradiated region

Metastases to the lymph nodes were diagnosed in 16 patients (8 in Group FB and 8 in Group BB, respectively). In 12 cases metastatic lesions were observed in the regional lymph nodes (5 in Group FB and 7 in Group BB, respectively) and in 4 cases in distant ones (3 in Group FB and 1 in Group BB, respectively).

Overall, the 5-year freedom from incidence of distant metastases (lymph nodes/bones) was 85%, with no statistical differences between the FB and BB Groups (*p* = 0.31). Factors which influenced the time duration to the occurrence of metastases in the univariate analysis were: T-stage (*p* = 0.014), the Gleason score (*p* = 0.000..), the highest PSA concentration before RT (*p* = 0.018) and the risk group (*p* = 0.003). In the multivariate analysis, the Gleason score was the only significant factor (*p* = 0.005). 

### Follow-up: Biochemical control

Biochemical recurrence was observed in 26 patients. The 5-year biochemical control was 83.5% in the FB Group and 87.6% in Group BB (*p* = 0.14).

### Toxicity

RT was well tolerated in both groups. The incidence of acute ≥G2 GU toxicity was similar between groups: 39 cases in Group BB vs 42 in Group FB (*p* = 0.75). However, more patients in the BB group experienced severe Grade 3 toxicity (*p* = 0.035). The incidence of acute ≥G2 GI toxicity was also similar: 25 cases in Group FB vs 19 in Group BB (*p* = 0.2). However, in the FB group statistically more patients did not experience any GI toxicity (*p* = 0.019). The actuarial 5-year incidence of late G2+ GU toxicity was 12% in both groups (*p* = 0.98) and G2+ GI toxicity 19% vs 15% (*p* = 0.55), respectively. Detailed characteristics are presented in Table 5.

**Table 5:** Treatment toxicity

Bone – based image guided group (BB) *N* = 89		Fiducial – based image guided group (FB) *N* = 91		
GI	Number (%)	GI	Number (%)	*p*
Acute: during treatment and the first 3 months of follow-up	89 (100%)	Acute: during treatment and the first 3 months of follow-up	91 (100%)	0.29
G0	26 (29.2%)	G0	40 (43.9%)	
G1	38 (42.7%)	G1	32 (35.2%)	
G2	24 (27.0%)	G2	18 (19.8%)	
G3	1 (1.1%)	G3	1 (1.1%)	
Late: after the third month of follow-up	89 (100%)	Late: after the third month of follow-up	88 (96.7%)	0.90
G0	51 (57.3%)	G0	53 (60.2%)	
G1	26 (29.2%)	G1	20 (22.8%)	
G2	9 (10.1%)	G2	11 (12.5%)	
G3	3 (3.4%)	G3	4 (4.5%)	
GU	Number (%)	GU	Number (%)	p
Acute: during treatment and the first 3 months of follow-up	89 (100%)	Acute: during treatment and the first 3 months of follow-up	91 (100%)	0.33
G0	18 (20.2%)	G0	22 (24.2%)	
G1	32 (36.0%)	G1	27 (29.7%)	
G2	34 (38.2%)	G2	41 (45.0%)	
G3	5 (5.6%)	G3	1 (1.1%)	
Late: after the third month of follow-up	89 (100%)	Late: after the third month of follow-up	88 (96.7%)	0.12
G0	62 (69.7%)	G0	50 (56.8%)	
G1	16 (18.0%)	G1	27 (30.7%)	
G2	10 (11.2%)	G2	10 (11.4%)	
G3	1 (1.1%)	G3	1 (1.1%)	

At the beginning of radiotherapy, 167 patients (93%) answered the question on sexual activity with 53 of them (32%) further answering details concerning sexual functioning. After a 3-year follow-up, these rates were: 127 patients (71%) and 42 patients (33%). A total of 77 patients (43% of all patients and 46% of respondents) reported some sexual activity (at least a little) before RT as compared to 62 patients (34% of all pts. and 49% of respondents after 3 years of follow-up). The differences were not significant (*p* = 0.61). Pronounced erectile dysfunction (quite a bit or very much) was reported by 22 and 16 patients before and 3 years post RT (29 and 26% of those reporting sexual activity). The differences were not significant (*p* = 0.69). No significant differences between the BB and FB Group was observed either with respect to sexual activity before radiotherapy and at the 3-year follow up. Before radiotherapy some sexual activity was reported by 31 (40% respondents) in the BB Group and 46 patients (52% of respondents) in the FB Group, with pronounced sexual dysfunction presented in 9 (29% of sexually active) in the BB Group and 13 (28% of sexually active) in the FB Group (*p* = 0.1 and *p* = 0.92). At the 3-year follow-up, the rates were: 28 patients (42% of respondents) and 5 (18% sexually active) in the BB Group and 34 (57% of respondents) and 11 (32% sexually active) in the FB Group (*p* = 0.094 and *p* = 0.21).

## Discussion

There are only a few articles concerning the comparison of different IGRT techniques in terms of long-term treatment effects in prostate cancer patients [16–20]. Zelefsky M. et al. evaluated the results of 376 prostate patients who received IMRT with (186 patients) or without (190 patients) fiducial-based IGRT. In their study, no differences in bPFS outcomes were observed for LR and IR patients when treated with IGRT and non-IGRT, although HR patients treated with IGRT had significant improvement in bPFS 3 years post-treatment [16]. Valeriani M. et al. evaluated the efficacy of 3D conformal hypofractionated RT with or without IGRT in 106 IR prostate cancer patients. A kV cone beam CT was administered to 69 of them daily. With a median FU of 31 months, the 3-year OS and bPFS were 95 and 94%, respectively, with no differences between the IGRT and non-IGRT group [17]. Sveistrup J. et al. compared the RT results of HR prostate cancer patients treated with 3DCRT (115 patients, total dose of 76 Gy) and fiducial based IG-IMRT (388 patients, total dose of 78 Gy). The groups differed not only in terms of treatment techniques, daily imaging, total dose, but also in the PTV margins which were 1–2 cm in the 3DCRT group and 5–7 mm in the IG-IMRT group. Despite these differences, the 3-year bPFS was 86% for 3DCRT and 90% for IG-IMRT with non-significant statistical results [18]. Singh J. et al. evaluated the results of 282 patients (154 in the fiducial – based IGRT group) with a follow-up between 8 to 26 months. In the IGRT group patients were more frequently treated with a higher than 70 Gy dose, but the CTV-PTV margins were significantly smaller [19]. Gil S. et al. described data concerning 275 patients before (26 patients) and after (249 patients) the implementation of a fiducial marker IGRT program. Moreover, in their study, the dose was escalated from 74Gy to 78Gy [17]. Singh J. and Gill S. in their studies did not report a treatment related outcome in terms of survival or biochemical control [19, 20]. Our results showed that IGRT with a fiducial did not improve bPFS as compared to bone-based positioning. However, we observed that fiducial-based IGRT marginally improved the OS rate in patients with IR prostate cancer, which is in contrast to the results seen in the study by Zelefsky et al. in which they noted a benefit from IGRT only in the HR group [16]. We concluded that those who are at a lower risk of metastatic spread might benefit more from an accurate local treatment than those who are already at a high risk. The longer follow-up in our study than in Zelefsky et al. (median FU of 5.0 vs. 2.8 years) might suggest which groups of patients benefit more from the use of IGRT, but still further observations are needed as the difference did not reach statistical significance. Although, the distribution of clinical factors was not statistically different between the BB and FB groups, we cannot exclude some influence by a slight imbalance in factors such as the Gleason score, which proved to be an important prognostic factor.

What was noticeable in all the above-mentioned studies was the fact that IGRT reduced treatment-related toxicity. Zelefsky et al. in their study observed a 3-year likelihood of G2+ GU toxicity of 10% in the fiducial-based group compared to 20% in the no-IGRT group [16]. Moreover, Valeriani et al. observed a lower incidence of G2+ late rectal toxicities in the IGRT group (1.6% vs. 14.5%, *p* = 0.021) [17]. A very high difference in the 2-year likelihood of developing G2+ GI toxicity was observed in the study by Sveistrup et al. [18]: 57.3% in 3DCRT patients and 5.8% in IG-IMRT patients (*p* < 0.001). This might be a result of not only the implementation of IGRT but, what is more likely, a very steep decrease in the PTV margins – from 1 to 2 cm to only 5–7 mm. In the study by Singh J.et al. [19], patients were asked to complete postal questionnaires describing their bowel and bladder symptoms. Improvement was noted in all dysfunctional rectal symptoms (pain, urgency, diarrhea, bowel habit changes) in the IGRT group - with similar urinary symptoms in both groups. Gill et al. also observed a reduction in ≥G3 urinary frequency (23% vs. 7%, *p* = 0.018), ≥G2 diarrhea (15% vs. 3%, *p* = 0.017) and ≥ G2 fatigue (23% vs. 8%, *p* = 0.027) after implementation of IGRT. Furthermore, the median number of days with any toxicity was higher in the non-IGRT group [20]. These observations were not confirmed in our study - however, this might be due to: the relatively small margins used in our institution, no special margin reduction with FB IGRT and strict constraints for OARs irrespective of the IGRT method. What is important, contrary to many of the studies which compared IGRT to no IGRT, is that we compared two forms of IGRT: a more sophisticated FB and the basic one – BB. It could influence the results indicating only a slight impact of the IGRT method on the treatment outcome.

Radiotherapy may negatively influence sexual functioning of patients, especially causing erectile dysfunction, and it is suggested that this fact is associated with the dose delivered to the penile bulb [23, 24]. However, this seems to be less pronounced in modern radiotherapy techniques than with older ones. In our study we did not observe significant deterioration in sexual functioning as compared to the pre-RT status. Nor were there significant differences between the BB and FB Groups. The lack of difference when comparing to the pre-RT stage may seem questionable, however the deterioration of sexual activity in some patients could be counterbalanced by a lack of hormonal blockade in those patients who did not continue adjuvant HT. However, in our opinion, the reporting on sexual function by our patients may not be quite reliable as 43% of patients reported some sexual activity while 88% were on neoadjuvant hormonal therapy. Additionally, among the patients who responded to the question regarding sexual activity only approximately 30% of them responded to detailed questions regarding sexual function including erectile functioning or dysfunction. The latter could, however, be also attributable to the negative attitudes of our patients in describing details of sexual intimacy. Nonetheless, the results of the present analysis on sexual functioning should be interpreted with caution due to potential patient-related bias.

There are, some drawbacks of fiducial based IGRT performed in our center: the implantation of only one fiducial and a lack of tracking during the treatment session. Three fiducials are considered a more standard IGRT procedure. Perhaps changing our policy in this field could improve the accuracy of IGRT and thus treatment outcome. Introduction of a real-time kilovoltage intrafraction monitoring system which tracks the target motions in translational and rotational directions during treatment could further provide more satisfactory results [25]. It should also be noted that we used slightly lower total doses than recommended by the latest trials in the intermediate and high risk groups.

## Conclusions

Despite some benefit in terms of acute toxicity in the FB group, when comparing bone-based to fiducial-based techniques, no differences in clinical outcomes or late toxicity were seen in this population. However, intermediate risk prostate cancer patients are those who might benefit the most from the implementation of fiducial-based IGRT. Further studies with a longer follow-up are needed to confirm this hypothesis.

## Abbreviations

BB: Bone-based
CT: Computed tomography
CTV: Clinical target volume
CVD: Cardiovascular diseases
FB: Fiducial-based
FU: Follow-up
HR: High risk (group)
HT: Hormonal therapy
IGRT: Image-guided radiotherapy
IMRT: Intensity modulated radiotherapy
IR: Intermediate risk (group)
kV: Kilovoltage
LR: Low risk (group)
OARs: Organs at risk
OS: Overall survival
PFS: Progression-free Survival
PSA: Prostate specific antigen
PTV: Planning target volume
RT: Radiation therapy
TD: Total dose

## References

[1] Gómez-Millán J, Lara MF, Correa Generoso R, Perez-Rozos A, Lupiáñez-Pérez Y, Medina Carmona JA (2015). Advances in the treatment of prostate cancer with radiotherapy. Crit Rev Oncol Hematol.

[2] Ten Haken RK, Forman JD, Heimburger DK, Gerhardsson A, McShan DL, Perez-Tamayo C (1991). Treatment planning issues related to prostate movement in response to differential filling of the rectum and bladder. Int J Radiat Oncol Biol Phys.

[3] Nabavizadeh N, Elliott DA, Chen Y, Kusano AS, Mitin T, Thomas CR, Holland JM (2016). Image guided radiation therapy (IGRT) practice patterns and IGRT's impact on workflow and treatment planning: results from a National Survey of American Society for Radiation Oncology members. Int J Radiat Oncol Biol Phys.

[4] De Los Santos J, Popple R, Agazaryan N, Bayouth JE, Bissonnette JP, Bucci MK (2013). Image guided radiation therapy (IGRT) technologies for radiation therapy localization and delivery. Int J Radiat Oncol Biol Phys.

[5] Das S, Liu T, Jani AB, Rossi P, Shelton J, Shi Z, Khan MK (2014). Comparison of image-guided radiotherapy technologies for prostate cancer. Am J Clin Oncol.

[6] Fraass BA, Jolly S, Eisbruch A. Conformal Therapy and Intensity- Modulated Radiation Therapy: Treatment planning, treatment delivery, and clinical results. In: clinical radiation oncology, 3^rd^ ed. Gunderson LL, Tepper JE, Philadelphia 2012; 15: 287–316.

[7] Tanyi JA, He T, Summers PA, Mburu RG, Kato CM, Rhodes SM (2010). Assessment of planning target volume margins for intensity-modulated radiotherapy of the prostate gland: role of daily inter- and intrafraction motion. Int J Radiat Oncol Biol Phys.

[8] Chung HT, Xia P, Chan LW, Park-Somers E, Roach M (2009). Does image-guided radiotherapy improve toxicity profile in whole pelvic-treated high-risk prostate cancer? Comparison between IG-IMRT and IMRT. Int J Radiat Oncol Biol Phys.

[9] Rudat V, Nour A, Hammoud M, Alaradi A, Mohammed A (2016). Image-guided intensity-modulated radiotherapy of prostate cancer: analysis of interfractional errors and acute toxicity. Strahlenther Onkol.

[10] Khor R, Williams S (2013). Contemporary issues in radiotherapy for clinically localized prostate cancer. Hematol Oncol Clin North Am.

[11] Kupelian PA, Langen KM, Willoughby TR, Zeidan OA, Meeks SL (2008). Image-guided radiotherapy for localized prostate cancer: treating a moving target. Semin Radiat Oncol.

[12] Miszczyk L, Leszczyński W, Szczepanik K, Majewski W (2008). Comparison of two image guided radiation therapy (IGRT) methods used for prostate cancer patients - CBCT and 2D-2D kV. Przeglad Lekarski.

[13] Langen KM, Willoughby TR, Meeks SL, Santhanam A, Cunningham A, Levine L, Kupelian PA (2008). Observations on real-time prostate gland motion using electromagnetic tracking. Int J Radiat Oncol Biol Phys.

[14] Goyal S, Kataria T (2014). Image guidance in radiation therapy: techniques and applications. Radiol Res Pract.

[15] Dang A, Kupelian PA, Cao M, Agazaryan N, Kishan AU (2018). Image-guided radiotherapy for prostate cancer. Transl Androl Urol.

[16] Zelefsky MJ, Kollmeier M, Cox B, Fidaleo A, Sperling D, Pei X (2012). Improved clinical outcomes with high-dose image guided radiotherapy compared with non-IGRT for the treatment of clinically localized prostate cancer. Int J Radiat Oncol Biol Phys.

[17] Valeriani M, Bracci S, Osti MF, Falco T, Agolli L, De Sanctis V, Enrici RM (2013). Intermediate-risk prostate cancer patients treated with androgen deprivation therapy and a hypofractionated radiation regimen with or without image guided radiotherapy. Radiat Oncol.

[18] Sveistrup J, af Rosenschöld PM, Deasy JO, Oh JH, Pommer T, Petersen PM, Engelholm SA (2014). Improvement in toxicity in high risk prostate cancer patients treated with image-guided intensity-modulated radiotherapy compared to 3D conformal radiotherapy without daily image guidance. Radiat Oncol.

[19] Singh J, Greer PB, White MA, Parker J, Patterson J, Tang CI (2013). Treatment-related morbidity in prostate cancer: a comparison of 3-dimensional conformal radiation therapy with and without image guidance using implanted fiducial markers. Int J Radiat Oncol Biol Phys.

[20] Gill S, Thomas J, Fox C, Kron T, Rolfo A, Leahy M (2011). Acute toxicity in prostate cancer patients treated with and without image-guided radiotherapy. Radiat Oncol.

[21] Cox JD, Stetz J, Pajak TF (1995). Toxicity criteria of the radiation therapy oncology group (RTOG) and the European Organization for Research and Treatment of Cancer (EORTC). Int J Radiat Oncol Biol Phys.

[22] van Andel G, Bottomley A, Fosså SD, Efficace F, Coens C, Guerif S (2008). An international field study of the EORTC QLQ-PR25: a questionnaire for assessing the health-related quality of life of patients with prostate cancer. Eur J Cancer.

[23] Fiorino C, Valdagni R, Rancati T, Sanguineti G (2009). Dose-volume effects for normal tissues in external radiotherapy: pelvis. Radiother Oncol.

[24] Cozzarini C, Rancati T, Badenchini F, Palorini F, Avuzzi B, Degli Esposti C (2016). Baseline status and dose to the penile bulb predict impotence 1 year after radiotherapy for prostate cancer. Strahlenther Oncol.

[25] Nguyen DT, O'Brien R, Kim JH, Huang CY, Wilton L, Greer P (2017). The first clinical implementation of a real-time six degree of freedom target tracking system during radiation therapy based on Kilovoltage Intrafraction monitoring (KIM). Radiother Oncol.

